# Titanium Oxide and Zinc Oxide Nanoparticles in Combination with Cadmium Tolerant *Bacillus pumilus* Ameliorates the Cadmium Toxicity in Maize

**DOI:** 10.3390/antiox11112156

**Published:** 2022-10-31

**Authors:** Tayyab Shafiq, Humaira Yasmin, Zafar Abbas Shah, Asia Nosheen, Parvaiz Ahmad, Prashant Kaushik, Ajaz Ahmad

**Affiliations:** 1 Department of Biosciences, COMSATS University Islamabad (CUI), Islamabad 45550, Pakistan; tayyabshafiqe@gmail.com (T.S.); asianosheen@yahoo.com (A.N.); 2 Department of Bioinformatics, Hazara University, Mansehra 21120, Pakistan; zafarabbasshah92@gmail.com; 3 Department of Botany, GDC Pulwama, Pulwama 192301, Jammu and Kashmir, India; 4 Independent Researcher, 46022 Valencia, Spain; prashantumri@gmail.com; 5 Department of Clinical Pharmacy, College of Pharmacy, King Saud University, Riyadh 11451, Saudi Arabia; ajukash@gmail.com

**Keywords:** cadmium stress, titanium oxide nanoparticles (NPs), zinc oxide nanoparticles (ZnO NPs), antioxidant, micronutrients, in silico analysis

## Abstract

The efficiency of Cd-tolerant plant growth-promoting bacteria (PGPB), zinc oxide nanoparticles (ZnO NPs), and titanium dioxide nanoparticles (TiO_2_) in maize growing in Cd-rich conditions was tested in the current study. The best Cd-tolerant strain, *Bacillus pumilus*, exhibited plant growth stimulation in vivo and in vitro experiments. We determined the toxic concentrations (30 (ppm)) of both NPs for plant growth. *B. pumilus*, ZnO NPs (20 (ppm)), and TiO_2_ NPs (10 (ppm)) had a synergistic effect on plant growth promotion in Cd-contaminated soil (120 (ppm)) in a pot experiment. Both alone and in combination, these therapies reduced Cd toxicity, resulting in improved stress metabolism and defense responses. The combined treatments showed increased relative water content, photosynthetic pigments, proline, total sugars, and proteins and significantly reduced lipid peroxidation. Moreover, this combination increased the levels of minerals and antioxidants and reduced Cd bioaccumulation in shoots and roots by 40–60%. Our in silico pipeline presented a novel picture of the participation of ZnO–TiO_2_ protein interaction in both *B. pumilus* and maize. These findings provide fresh insights on the use of *B. pumilus*, ZnO, and TiO_2_ NPs, both separately and in combination, as a viable and environmentally benign strategy for reducing Cd stress in maize.

## 1. Introduction

Worldwide maize production reached 1162 million tons in 2020. The worldwide output of maize increased from 313 million tons in 1971 to 1162 million tons in 2020, expanding at a 3.07% yearly pace [1]. The United States acquired 12.1 million tons and 11.31 million tons, respectively [2]. Pakistan’s alarming population growth rate has exacerbated the country’s food shortages. During the previous two decades, increased cereal output has been countered by a 3% growth in the human population. Irrigation is available for around 66% of maize production [3,4].

Metal contamination is a serious hazard for soil and cadmium (Cd) is a non-essential and hazardous heavy metal that is readily taken up by roots and deposited in diverse plant tissues, posing a global threat to crop development and output. Plants use various mechanisms to resist Cd’s inhibitory impact, including nutrient management as one option for overcoming Cd toxicity [5]. Sulfur (S) absorption and assimilation are critical for crop productivity and Cd stress tolerance. Cd impacts the S assimilation route, which results in the activation of the cysteine (Cys) biosynthesis pathway, which is a precursor to glutathione (GSH) production [6]. Cd is a dangerous heavy metal that may injure humans, animals, and plants. It mainly enters agricultural soils via industrial processes, phosphate fertilizers, and atmospheric deposition, then goes up the food chain [7,8]. 

Cadmium (Cd) is a hazardous heavy metal; nevertheless, its toxicity varies depending on the organism. Cadmium-tolerant bacteria (CdtB) are varied and are not linked phylogenetically. Because of their ecological significance, bacteria become more significant when pollution occurs, and human health is affected. Exploring plant-growth-promoting rhizobacteria in agriculture is thus one of the more promising approaches for enhancing agricultural yield while minimizing environmental damage. Simultaneously, nanotechnological applications have a significant impact [9]. Plant-growth-promoting rhizobacteria can act as helpers to induce the germination of seeds, and PGPR can promote plant growth throughout the plant’s life. Rhizobacteria are found in soil and they have symbiotic relationships with the plant [10]. Biofertilizers containing rhizobacteria can improve crop yields, especially that of maize [11].

Novel technologies are frequently used to attain the requisite crop yields in the quest to increase food production with an environmentally responsible approach and with limited resources. Compared to conventional fertilizers, nanofertilizers are more successful in promoting plant development because plants quickly absorb them. As a result, nanomaterials such as nanofibers, nanofertilizers, and nanopesticides have the potential to revolutionize agriculture. With nanomaterials such as silver, titanium, zinc oxide, silica, and gold, PGPR could be a promising technique for controlling plant growth and productivity. To investigate interactive studies of PGPR with different nanoparticles in a cost-effective and result-oriented manner, a prediction-based validation method was utilized to lower the cost of experimentation [12]. On the other hand, engineered metal nanoparticles can have beneficial and harmful effects on rhizobacteria [13].

Nanotechnology has paved the way towards many applications, and the introduction of nanoparticles such as metallic nanoparticles into the biological sciences has been noted to provide desired effects. In addition to this hese particles having been used in environmental cleanup and soil transport systems. The size, shape, inertness, and surface coating materials of nanoparticles are well-known. Under certain conditions, they are less hazardous and highly resistant to surface oxidation. Furthermore, nanoparticles are delivered into the cell using different routes depending on their size. PGPR are soil microorganisms, which are regarded as ecosystem building blocks, and are vulnerable to nanoparticles, although their mechanisms of action are unknown. This could be due to nanoparticles’ various types and natures, and there is no specific model for interpreting nanoparticle interactions with PGPR [14].

Different plants have benefited from the application of titanium dioxide (TiO_2_) and zinc oxide nanoparticles (ZnO), which reduced soil salinity. NPs have also made an impact on sustainable agriculture. Furthermore, they have been applied to the roots or as a foliar spray to boost plant growth enzyme activity, chlorophyll content, photosynthesis, nutrient absorption, stress tolerance, yield, and crop quality. Agricultural products which can be produced from nanoparticles are crucial for increasing plant growth and crop yield. In addition, they provide favorable alternatives for farmers, compared with conventional chemicals and techniques, because of various properties such as their small size, simplicity of transport, easy handling, long-term storage, high efficacy, and nontoxicity. As a result, nano-based commercialization is gaining popularity worldwide [15].

The main aim of this study was to illustrate the application of TiO_2_ and ZnO NPs with PGPR to tackle the toxic effect of Cd stress on soil and its accumulation in plants, as well priming seeds with PGPR and NPs to increase the yield of maize. The best treatment was found to be the combined use of PGPR and TiO_2_ and ZnO NPs. However, these results can only be practically applied with the use of the appropriate PGPR strains and appropriate concentrations of TiO_2_ and ZnO NPs. Different concentrations can harm soil and plant as well. Further research is needed to evaluate and quantify the mode of action of NPs and PGPR, which hinder the impact of heavy metals in soil and plants.

## 2. Materials and Methods

### 2.1. Collection of Seeds 

Fresh seeds of the maize variety “Sargodha gold” were taken from the National Research Centre (NARC), Islamabad, Pakistan. First, the surface was sanitized with a 90% ethanol solution, before being treated with a 3.5% sodium hypochlorite solution. After that, the seeds were washed five times with distilled water [16].

### 2.2. Evaluation of the Toxicity of TiO_2_ and ZnO NPs on Maize Seeds

TiO_2_ and ZnO NPs were evaluated to determine their toxic concentrations. Four different concentrations—25, 50, 100, and 150 ppm—were used. Soaked seeds were then analyzed to determine the effect of varying concentrations of NPs.

### 2.3. Screening of Cd-Tolerant Strains 

Bacterial strains (CM1, RH10, RHG, MRH1, CM2, 9K, CM5, BG, JW, 18RKI) preserved in glycerol were taken from the Applied Microbiology and Biotechnology Lab, COMSATS University Islamabad, Islamabad. To select the bacterial strain with the highest cadmium (Cd) tolerance, the ten bacterial strains were allowed to grow on Petri plates for 48 h with various Cd concentrations, i.e., 2 ppm, 4 ppm, 6 ppm, and 8 ppm. Seeds were soaked in bacterial pellets for 3 h for this purpose. Pellets were prepared by obtaining the pure cultures of strains and then allowing them to grow in a liquid broth. After 24–48 h, each culture was centrifuged for 15 min at 4000 rpm to obtain the desired pellets. We aimed to determine the most Cd-tolerant strain by determine root length (RL), shoot length (SL), leaf area (LA), fresh weight (FW), and dry weight (DW).

### 2.4. Selection of the Cd Concentration for the Germination of Seeds

To select a tolerable concentration of Cd for the seeds of maize, the sterile seeds of maize were grown on Petri plates in triplicates at different concentrations (40 ppm, 80 ppm, and 120 ppm) along with the control which had no Cd stress. Seeds were allowed to grow for two weeks; after that, their germination and biomass were noted.

### 2.5. Pot Experiment

A pot experiment was conducted in the greenhouse of COMSATS University, Islamabad (33.7294° N, 73.0931° E, average temperature = 15 °C, humidity = 64%). The experiment was conducted by ensuring the availability of natural light and 1.5 kg of autoclaved dried soil enriched in potassium and nitrogen. The soil which was used was called nitisol (WRB 2015) and the predominant soil particles were fine sand and silt, with percentages ranging from 49.48% to 77.78% and 10.66% to 34.23% (3:1, soil:sand). Soil was placed in pots of 10 × 10 cm in size with drainage holes in the bottom. A plastic plate was placed under each pot to collect the leachate, which was further irrigated onto the soil surface. Eight seeds per pot were sown in the autoclaved soil. Each treatment was applied in triplicate. The moisture of the soil content was maintained around 60% with distilled water. The finalized doses of TiO_2_ and ZnO NPs (20 ppm) and cadmium stress were applied to the plants at the three-leaf stage. After 15 days of treatment, the plants were harvested. The experimental design was randomized, and the specifics are listed below. For biomass analyses, root and shoot lengths, fresh and dry weights, leaf area, and relative water contents were separated. Roots and shoots were harvested separately for each duplicate and kept at 40 °C for various enzymatic tests.

### 2.6. Biomass Studies of Maize

Biomass studies involve the assessment of parameters such as the shoot length, root length, fresh weight, relative water content, dry weight, germination percentage, germination index, promptness index, and seedling vigor index. The harvested plants were measured using a measuring scale and a weighing balance.

### 2.7. Relative Water Content

To determine the RWC, leaves were soaked in water overnight after measuring their fresh weight. The next day, the fully turgid weight of maize leaves was measured. The leaves were then allowed to dry to determine the dry weight [16,17].

Percentage of RWC = [(Fresh weight − dry weight)/(fully turgid weight − dry weight)] × 100

### 2.8. Evaluation of Photosynthetic Pigments

The estimation of photosynthetic pigments such as carotenoids and chlorophyll a and b was performed by placing 0.5 g of the fresh leaves in test tubes. Into each test tube, 10 mL of dimethyl-sulfoxide (DMSO) was added and the test tube was allowed to stay for 4 h in the water bath at 65 °C or for 72 h at room temperature. After this period, the supernatant was taken, and the absorbance was measured at 480, 648, and 665 nm. All of the calculations were performed according to the protocol of Chappelle, Kim [18].

Estimation of carotenoids = OD 480 × 4

Estimation of chlorophyll a (mg/g) = [12.7(OD 665) − 2.69 (OD 648)] × V/1000 × W Estimation of chlorophyll b (mg/g) = [22.9(OD 648) − 4.68 (OD 665)] ×V/1000 × W

#### 2.8.1. Preparation of Enzyme Extract

To prepare the enzyme extract, 0.5 g of roots and leaves were taken separately and washed with distilled water. The roots and leaves were ground on a pre-cooled pestle and mortar and 2 to 3 mL of phosphate buffer was added, ensuring that the pH was 7 to 8. A homogenized sample was made, and the volume of the solution was raised to 5 mL. The samples were centrifuged at 8000 to 13,000 rpm at 4 °C for 15 min [19].

#### 2.8.2. Evaluation of Proline Content

The protocol reported by Gibon [20] was followed to quantify the proline content. Fresh leaves (0.5 g) of maize were taken and homogenized using 80% ethyl alcohol. Leaves were further processed for the water bath treatment at 80 °C for 1 h. Centrifugation was performed to obtain 0.5 mL of the supernatant leaf extract, which was then moved into another test tube. Then, 0.5 mL of distilled water and 5% of phenol (1 mL) were added to the supernatant and placed for 1 h in the incubator. After incubation, absorbance was checked at 490 nm by adding 2.5 mL of sulfuric acid.

T. SS = sample absorbance × K.value × D.F/sample weight × 100

where

D.f = final volume/initial volume

#### 2.8.3. Lipid Peroxidation Test, Evaluation of Malondialdehyde

The quantity of lipid peroxidation was calculated based on the malondialdehyde (MDA) generated by thiobarbituric acid in this test. The technique developed by Buege and Aust [20] was followed here. We cut up a plant sample (0.5 g) and rinsed it with distilled water. Over ice, samples were pulverized in a pestle and mortar. The samples were homogenized in 100 mM phosphate buffer (pH 8), and the total volume was 8 mL. The samples were centrifuged at 4 °C for 15 min 8000–13,000 rpm.

MDA (nmol/g FW) = (OD 532 − OD 600) (A × v) (a × E × w) A = Reaction solution + used enzyme extract

#### 2.8.4. Estimation of Antioxidant Enzymes

The catalase (CAT) activity was assessed using the procedure of Kumar et al. [21], and the absorbance was measured at 240 nm at the 0-time interval and after 3 min. The activity of ascorbate peroxidase (APX) was determined using the procedure of Rao et al. [22]. The absorbance was measured at 290 nm at 0 and 3 min. The peroxide dismutase (POD) activity was assessed using a modified methodology developed by Onsa et al. [23]. At 420 nm, the absorbance was measured. A procedure developed by Lück [24] was used to estimate superoxide dismutase (SOD) activity. Two sets were observed for 20 min, one in 100% light at 4000 lux and the other in deep darkness.

### 2.9. Nutrient Analysis

Dried maize leaves (0.5 g) were subjected to acidic digestion [25]. The Cd and nutrient content of magnesium (Mg), potassium (K), zinc (Zn), calcium (Ca), copper (Cu), iron (Fe), and sodium (Na) were measured.

### 2.10. Construction of ZnO–TiO_2_ Protein Interaction Network

We used the STITCH version 5.0 database to construct the ZnO–TiO_2_ protein interaction network. The STITCH database is a knowledge base of interactions among proteins and small molecules. This database is widely used for determining inter-relationships between drug targets, chemicals/receptors, and single molecules associations with metabolic pathways. We entered the “ZnO-TiO_2_” compound as a query in either *B. pumilus* or *Zea may* L. species, which displayed a protein interaction network. The database also provided multiple functional tabs for further analysis and enrichment studies of a given network. Finally, we exported both ZnO–TiO_2_ protein interaction networks with a confidence score of 0.400 for the comprehensive coverage of available interactions.

### 2.11. Statistical Analysis

STATISTIX (version 8.1) software was used to evaluate all of the data statistically. To compare different mean values of all treatments, the least significant difference (LSD) was computed at *p* = 0.05 [26].

## 3. Results

### 3.1. Screening of Cd-Tolerant PGPB

These 10 bacterial strains (JW, 13RKI, GW3, BW, CM5, RHG MRH1, RH10, 9K, 8RK1) exhibited cadmium (Cd) tolerance. Out of these 10 bacterial strains, 6 of them showed morphological changes at different cadmium concentrations. At 2 ppm, strains showed colony formation, thus indicating resistance against Cd stress. By increasing the concentration from 4 to 6 ppm, the growth of each strain decreased. Only JW, 9K, and RHG, 13RK1 showed the maximum resistance against Cd stress of 6 ppm, which was depicted by enhanced growth on the media supplemented with Cd stress. Thus, these strains were selected for the seed priming of maize to further extract the best one, with the most prominent growth-inducing ability chosen as the treatment to be used in the pot experiments.

### 3.2. Maize Treatment with Cd-Tolerant PGPB

Maize showed a 40% germination rate without PGPR priming. However, the germination rate varied in different PGPR strains treatments. The maximum germination rate in the JW treatment was observed to be 85% compared to the control (without PGPR treatment). Other bacterial strains with effective germination-inducing abilities were 13RK1, GW3, 9K, BG, and MRH1. All the PGPR (13RK1, GW3, 9K, BG, and MRH1) showed a prominent increase in the shoot length (SL), root length (RL), and fresh weight (FW). The leaf area (LA) was promoted by all the PGPR strains except 8RK1 and RHG. Similarly, all the PGPR strain treatments showed an increase in the germination percentage (GP), germination index (GI), and promptness index (PI) in comparison with controls. However, the seedling vigor index (SVI) was promoted by all the PGPR strains except MRH1 and 8RK1. The maximum ability to increase all the parameters is shown by JW, which was selected for the pot experiments with maize plants. The maximum increase caused by JW in shoot length (SL), root length (RL), fresh weight (FW), leaf area(LA), dry weight (DW), germination percentage (GP), germination index (GI), productivity index (PI), and standard vegetation index (SVI) were revealed to be 33%, 195%, 300%, 430%, 600%, 113%, 128%, 116%, and 371%, respectively, compared with the controlled seedlings, as shown in Table 1. 

### 3.3. Cd-Tolerant Concentration for Maize

Cd stress (40, 80, and 120 ppm) induced severe damage to maize seedlings in the Petri plate experiment. Yellowing and wilting of leaves were the prominent features noted in the case of stressed seedlings compared with the control (without Cd stress). An increase in the doses of Cd stress decreased the SL, RL, LA, FW, DW, GP, GI, PI, and SVI by one, two, and three times compared to the control. Under the Cd stress level of 40 ppm, the distortion was not clearly visible, and the plant survived. However, the plant exhibited severe distortion under 120 ppm Cd stress. Thus, 120 ppm was regarded as the toxic Cd stress level for maize. Under 80 ppm Cd stress, the plants exhibited wilting, but the damage was not unbearable. Thus, the Cd concentration of 120 ppm was selected for the pot experiments.

### 3.4. Selection of Effective Concentrations of TiO_2_ and ZnO NPs for Maize

This Petri plate experiment involved TiO_2_ and ZnO NP treatments (10, 20, and 30 ppm) and showed that the most effective concentration of TiO_2_ and ZnO NPs was 20 ppm. The best growth results were observed at a concentration of 20 ppm for both NPs, but with the increase in the concentration NPs, wilting and yellowing of leaves were observed. The results from the experiment are shown in Table 2 and Table 3.

### 3.5. Pot Experiments

#### 3.5.1. Biomass Estimations

Cd stress (120 ppm) showed significant decreases in GP, GI, PI, and SVI of 75%, 30%, 35%, and 132% compared to the unstressed controlled plants. All the treatments showed significant stress alleviation. All the treatments involving seeds of maize primed with the JW strain revealed a substantial enhancement in the germination rate compared with unprimed seeds. Unprimed seeds exhibited a reduction in the germination rate of 20%. In unstressed plants, PGPR and NP treatments revealed a notable increase in the GP, GI, PI, and SVI, ranging from 9% to 44%. In the case of stressed plants the values ranged from 25% to 50%. In unstressed plants, combined treatment using PGPR and NPs exhibited maximum increases of 100%, 66%, 192%, and 212% in GP, GI, PI, and SVI, respectively. However, the maximum increase in the germination percentage, GI, PI, and SVI in the case of stressed plants exposed only to the JW treatment in combination with NP treatment was noted, with values of 39%, 41%, and 111% and 114%, 222%, and 260%, respectively, as shown in Table 4. 

#### 3.5.2. RWC, FW, and DW

Cd stress was associated with considerable decreases in plant biomass, i.e., FW, DW, and RWC, of 247.4%, 70%, and 22.2%, respectively, compared with non-stressed controls. A significant reduction in Cd-induced toxic effects was observed in the plant biomass in the case of all treatments. Plants treated with PGPR and NPs showed visible enhancements in FW, DW, and RWC, ranging from 21% to 76.7%, 81% to 122%, and 125% to 218%, respectively, in non-stressed maize plants. This increase ranged from 340% to 512%, 122% to 211%, and 111% to 233%, respectively, in stressed plants. All the treatments exhibited positive results in the maize seedlings even under stress. However, maximum increases of 86.7%, 142%, and 203% in FW, DW, and RWC, respectively, were revealed in the case of combined treatment with JW and NPS in non-stressed maize plants. On the other hand, in stressed plants, the maize seedlings exposed to NPs exhibited a maximum RWC value of 248%. Similarly, the maximum FW and DW values due to Cd stress alleviations were revealed under combined JW and NP treatment, at 582% and 239%.

### 3.6. Biochemical Estimations 

#### 3.6.1. Estimation of Chlorophyll and Carotenoid Content

**Figure 1:**
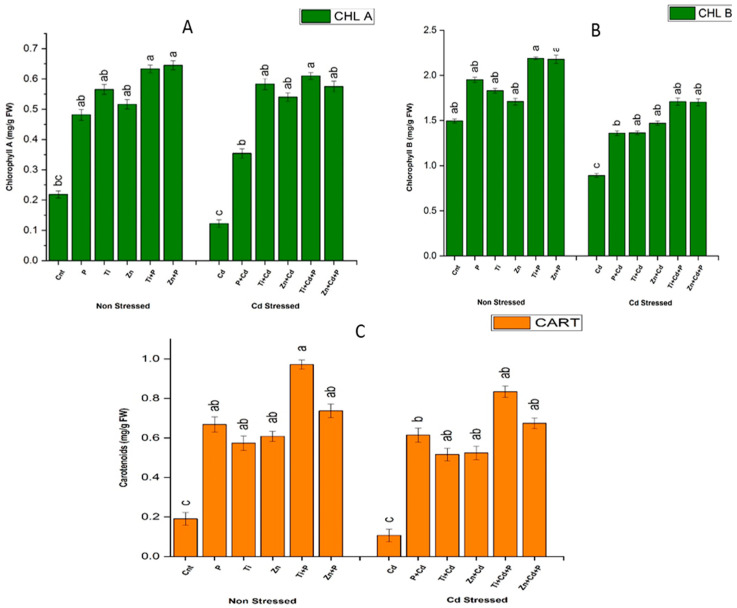
Effects of sole and combined treatments of plant-growth-promoting bacteria (PGPB) (*Bacillus pumilus*) and zinc oxide and titanium dioxide nanoparticles on (**A**) chlorophyll A, (**B**) chlorophyll B, and (**C**) carotenoid content. Cnt = non-treated, P = *Bacillus pumilus*, Ti = titanium dioxide nanoparticles, Zn = zinc oxide nanoparticles, Ti + P = titanium dioxide nanoparticles + *Bacillus pumilus* Zn + P = *Bacillus pumilus* + xinc oxide nanoparticles, Cd = cadmium stress, P + Cd = *Bacillus pumilus* + cadmium stress, Ti + Cd = *Bacillus pumilus* nanoparticles + cadmium stress, Zn + Cd = zinc oxide nanoparticles + cadmium stress, Ti + Cd + P = titanium dioxide nanoparticles + cadmium stress + *Bacillus pumilus*, Zn + Cd + P = zinc oxide nanoparticles + cadmium stress + *Bacillus pumilus*. To compare different mean values of all treatments, the least significant difference (LSD) was computed at *p* = 0.05.

Cd stress reduced the carotenoid and chlorophyll a and b content in maize by 42%, 65%, and 74.20%, respectively, compared with the non-stressed controls. All the treatments showed significant enhancements in carotenoid and chlorophyll a and b content in the non-stressed plants, ranging from 100% to 400%. In the case of stressed plants, all treatments showed elevations in carotenoid and chlorophyll a and b levels, ranging from 40% to 420%, respectively, compared with stressed controls. The maximum values of carotenoids and chlorophyll a and b were observed with the combined treatment of PGPR and NPs, with increases of 411%, 315%, and 65% in the case of non-stressed plants compared with non-stressed controls. Similarly, in the case of the Cd-stressed plants, the maximum Cd stress alleviation was observed with the combined treatment of PGPR and NPs, with increases of 502%, 238%, and 127% in the content of carotenoids and chlorophyll a and b, respectively (Figure 1).

#### 3.6.2. Estimation of Proline Content

**Figure 2:**
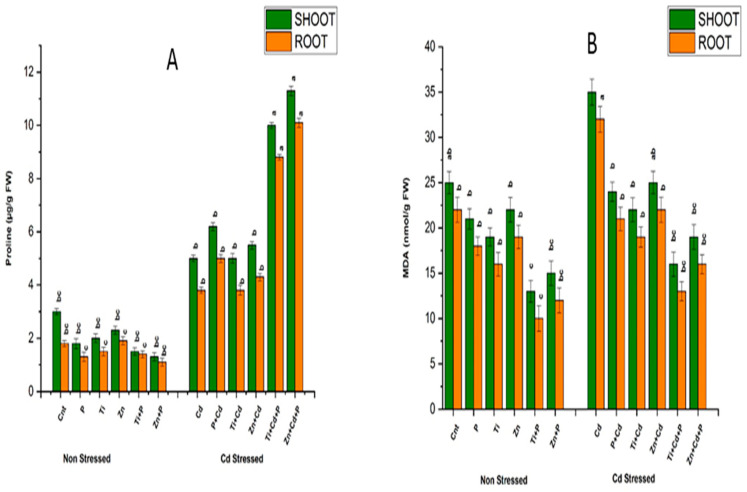
Effects of sole and combined treatments of plant-growth-promoting bacteria (PGPB) (*Bacillus pumilus*), zinc oxide, and titanium dioxide nanoparticles on (**A**) proline content (**B**) MDA content. To compare different mean values of all treatments, the least significant difference (LSD) was computed at *p* = 0.05.

The treatment of maize plants with Cd stress was associated with a significant increase in the proline content of 89.2%, compared with the non-stressed control. In the case of stressed plants, the application of PGPR and NPs compensated for the damage by producing more proline, with increases ranging from 3% to 300%. However, in non-stressed plants, the values of proline exhibited a significant reduction, ranging from 22% to 49% depicting the non-stressed state of plants. The maximum increase in proline content was observed with the combined treatment of PGPR and NPs, at 150% in the plants exposed to Cd stress. Similarly, in the case of non-stressed plants, the minimum proline content was detected in the combined treatment of PGPR and NPs, at 49%, compared with the non-stressed control. Proline helps combat damage in plants by protecting and enabling them to survive under stressful conditions (Figure 2).

#### 3.6.3. Estimation of Malondialdehyde (MDA)

Exposing plants to 120 ppm Cd stress resulted in severe damage to the lipid membrane, demonstrated by the enhanced values of MDA, which were 157% higher compared with non-stressed plants. However, the damage was reduced in exposed plants treated with PGPR and NPs, revealed by decreased MDA values ranging from 44% to 65% compared with the stressed plants. In the non-stressed plants, lower values of MDA ranged from 13% to 57%, revealing minor damage due to the non-stressed conditions compared with the non-stressed controls. With the combined treatment with PGPR we observed the maximum Cd stress alleviation, and NPs reduced the MDA value by 10%. However, in the case of non-stressed plants, the minimum MDA value was observed with the combined treatment with PGPR and NPs, which revealed a reduced MDA content of 59% compared to non-stressed control (Figure 2).

#### 3.6.4. Estimation of Catalase (CAT)

**Figure 3:**
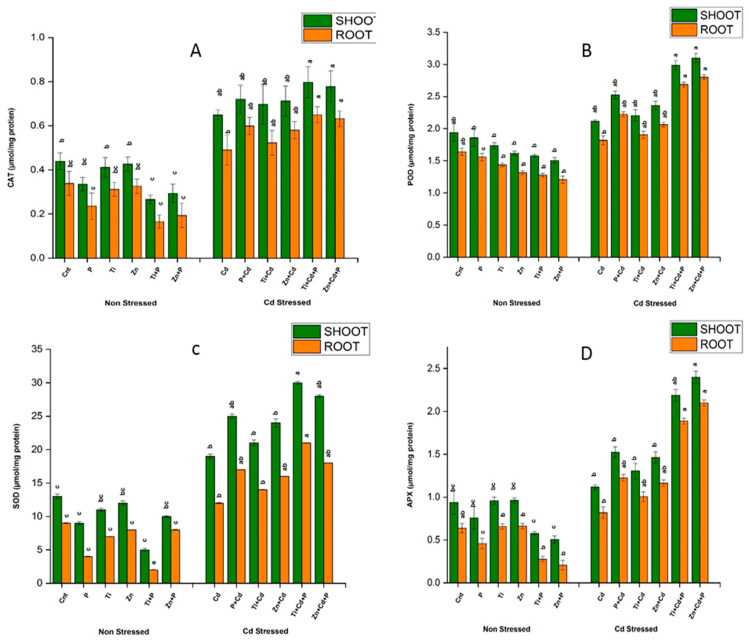
Effects of sole and combined treatments of plant-growth-promoting bacteria (PGPB) (*Bacillus pumilus*) and zinc oxide and titanium dioxide nanoparticles on (**A**) APX content, (**B**) CAT content, (**C**) POD content, and (**D**) SOD content. To compare different mean values of all treatments, the least significant difference (LSD) was computed at *p* = 0.05.

Compared to the control plants (without Cd stress), treating plants with 120 ppm Cd stress caused a rapid increase in CAT activity of 50% to 75% in leaves and roots, respectively. The level of CAT was further elevated in stressed plants due to PGPR and NP treatment, with increases ranging from 6% to 42% in leaves and 28% to 150% in roots, compared with stressed controls. In non-stressed plants, decreased values of CAT were observed, ranging from 20% to 51% and 23% to 53% in leaves and roots, respectively, compared with non-stressed controls representing the non-stressed state of *Zea mays*. The maximum CAT activity was observed in the combined treatment of PGPR and NPs, with increases of 45% and two times in leaves and roots, respectively, compared to the stressed controls. However, the minimum CAT activity was observed in non-stressed plants treated with the combined treatment of PGPR and NPs, at 41% and 43% in leaves and roots, respectively. The reduced CAT values observed in non-stressed conditions revealed the low concentrations of antioxidants due to the absence of stress exposure. Results depicting CAT activity are represented graphically in Figure 3.

#### 3.6.5. Estimation of Peroxidases (POD)

Exposing maize plants to Cd stress at 120 ppm raised the POD level in leaves and roots by 32% and 61%, respectively, compared with the non-stressed controls. Compared with the stressed plants, further treatment of stressed plants with PGPR and NPs boosted the POD levels in leaves and roots, with increases ranging from 22% to 81% and 4% to 90%, respectively. However, the maximum POD value was exceeded by 70% in the leaves and roots of stressed plants due to the synergistic treatment with PGPR and NPs. Moreover, non-stressed plants showed decreased POD values ranging from 16% to 61% and 21% to 52%, respectively, after the synergistic treatment with PGPR and NPs (Figure 3).

#### 3.6.6. Estimation of Superoxide Dismutase (SOD)

Maize plants with Cd stress exhibited 60% and 30% elevations in SOD compared with non-stressed plants. The treatment revealed a further increase in the SOD level in *Zea mays* with PGPR and NPs showing increases ranging from 20% to 70% in leaves and roots compared with the stressed controls. The maximum increase in the SOD value was observed in the combined treatment with PGPR and NPs, at 70% in the leaves and roots of maize. On the contrary, the level of SOD declined in the non-stressed plant and was as low as 10% to 500% in the non-exposed plants treated with the synergistic treatment containing PGPR and NPs compared with non-stressed plants (Figure 3).

#### 3.6.7. Estimation of Ascorbate (APX)

The APX level showed a rapid increase in the exposed maize plants with 120 ppm Cd stress, with increases of 15% to 40% compared to the non-exposed plants. In the exposed plants, the APX level was further increased by treatment with PGPR and NPs, with increases ranging from 20% to 250% and 18% to 210% in the leaves and roots, respectively, compared with stressed controls. On the contrary, in the case of non-stressed plants, the APX values showed a significant decline ranging from 10% to 220% in the leaves and roots compared to the non-stressed controls. However, the maximum APX value was revealed in the case of the combined treatment containing PGPR and NPs, at 250% and 220% in the leaves and roots of the stressed plants, respectively, compared with the stressed controls (Figure 3).

### 3.7. Nutrient Analysis

Cd stress was associated with a pronounced decrease in Fe, Mg, and Cu content compared to control and treated plants. However, maize plants showed a significant increase in Ca, Zn, K, Cd, and Na compared to controls but these were decreased compared to the treated plants. In NP-treated plants, the contents of Cu, Ca, Zn, K, Mg, Cd, Fe, and Na were increased by 100% to 458% compared to non-treated plants exposed to Cd stress.

There is a significant increase in the Cu, Ca, Zn, k, Mg, Cd, Fe, and Na content in NP-treated plants in the presence of *B. pumilus*, indicating that this was the best possible treatment for Cd-stressed plants. Furthermore, the maximum Cd remediation was exhibited by the synergistic action of PGPB and TiO_2_ and ZnO treatment, at 50–70% (Table 5).

### 3.8. ZnO–TiO_2_ Protein Interaction Network in B. pumilus

**Figure 4:**
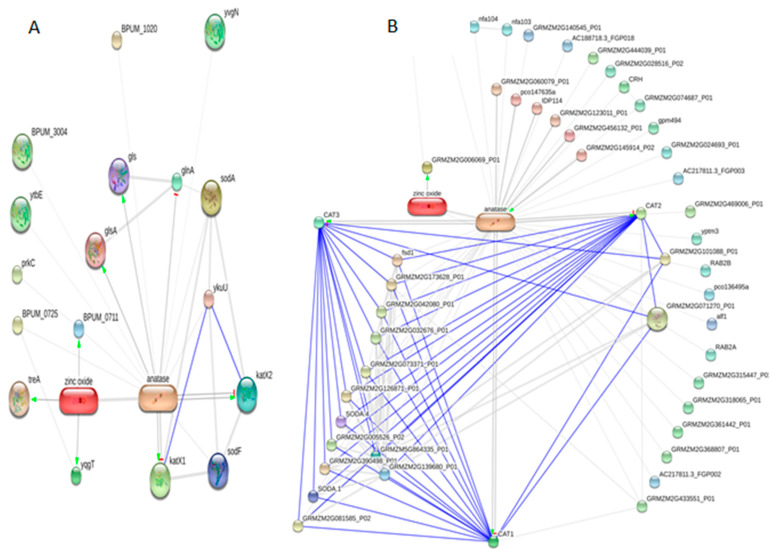
ZnO–TiO_2_ protein interaction pathway in *Bacillus pumilus* (**A**) ZnO–TiO_2_ protein interaction pathway in *Zea mays* (**B**).

We constructed a ZnO-TiO_2_ protein interaction network with 17 nodes (proteins) and 12 edges (interactions) in *B. pumilus*. ZnO positively regulates BPUM_0711, treA, and yqgT proteins, whereas TiO_2_ positively regulates glsA, katX1, KatX2, gls protein with the deactivation of the glnA protein. In this protein interaction network, several components, including BPUM_1020yvgN, sodA, ykuU, sodf, BPUM_0725, prkc, ytbE, and BPUM_3004, showed a lack of any physical association with the ZnO–TiO_2_ network. The network components were mainly involved in glyoxylate and dicarboxylate metabolism, microbial metabolism in diverse environments, D-glutamine and D-glutamate metabolism, and the alanine-aspartate-arginine-proline-tryptophan metabolism pathway. The network’s involvement in various pathways illustrates its role in carbohydrate synthesis from a fatty acid that leads to progress in growth and development under stress conditions (Figure 4).

### 3.9. ZnO–TiO_2_ Protein Interaction Network in Maize

We developed a ZnO–TiO_2_ protein interaction network with 50 nodes (proteins) and 83 edges (interactions) in maize. ZnO positively regulates GRMZM2G006069_P01, whereas TiO_2_ positively regulates IDP114, CAT1, CAT2, CAT3, and pco147635a, respectively, and the proteins GRMZM2G145914_P01, GRMZM2G456132_P01, and GRMZM2G121311_P01. The protein interaction network of the CAT1-CAT2-CAT3 complex exhibited, without a molecular mode of action, physical interaction with 15 members. The network components were mainly found to be involved in response to stimulus and oxidative stress, reactive oxygen species metabolism, hydrogen peroxide metabolism, and other diverse cellular event pathways. The network’s involvement in various pathways illustrates its role in plant survival, stability, and the rapid rational response to stress situations (Figure 4).

## 4. Discussion

Traditional methods such as soil cleaning, electrokinetics, vapor extraction, thermal desorption, and site management have been employed for a long time. However, traditional stress management methods are impractical, expensive, and time-consuming to implement. Furthermore, these approaches are responsible for disrupting native microbial communities in the soil, resulting in the emergence of more resistant species and secondary pollutants, increasing the expense and labor associated with sludge management [27]. In the present study, we opted for an ecofriendly technique to tackle heavy metal contamination in soil, affecting various plants and crops. The effect of Cd stress in maize was alleviated by applying PGPR and TiO_2_ and ZnO NPs.

PGPR strains are essential in allowing plants to adapt to new environments (both controlled and natural) [28]. In addition, they aid in the plant’s capacity to withstand HM stress by increasing the production of anti-stress hormones. Symbiotic interactions between PGPR and plants in the soil have been established as an effective technique for crop stress control, particularly in the HM detoxification process [29].

In this study, the detoxifying potential of PGPR (*B. pumilus*) and ZnO and TiO_2_ NPs in maize growing in Cd-rich soil was observed when they were used as single treatments and in combination. We discovered that Cd stress reduced GP and plant biomass in terms of SL, RL, LA, FW, and DW. The harmful effects of Cd on plants’ physiological and biochemical processes, the scarcity of nutrients, increased ROS production, and the breakdown of cellular machinery and chlorophyll contributed to the stunted development of plants and decreased biomass [30]. The accumulation of Cd caused a similar drop in the biomasses of various plants [31]. The use of PGPR and ZnO and TiO_2_ NPs resulted in significantly increased plant growth and biomass. Making use of symbiotic interactions between PGPR and plants in the soil has been recognized as a successful technique for managing stressed crops [32]. This increase in Cd resistance and improved plant growth and biomass were observed when applying both NPs, which delivered Zn and Ti to plants and regulated the synthesis of auxin, which is essential to the maintenance of cell membrane integrity under stress conditions [33]. This aids in restoring critical nutrients such as K, Ca, P, Mg, and Fe, which assist organelles such as mitochondria and chloroplasts in maintaining their structural and functional integrity. This leads to an improvement in plant defense mechanisms against ROS species. After using NPs and melatonin in soybean, effective Cd detoxification was observed [34].

We discovered that combining PGPR and NPs dramatically increased Cd resistance in maize plants. These findings are consistent with [35], who found that iron oxide NPs and PGPR worked together to reduce Cd toxicity in wheat effectively. Another study, presented in [36], revealed a substantial reduction in Cd toxicity and reduced Cd buildup in wheat. The immobilization of mobile HM factions resulted in a stress reduction through the use of nanotechnology [31]. ZnO and TiO_2_ NPs are a viable means of delivering Zn and Ti to plants. Zn is required for the regulation of IAA and auxin synthesis [37]. Under stressful situations, it is also critical in maintaining cell membrane integrity (Ref). It aids in the intake of essential nutrients such as K, Ca, P, Mg, and Fe [38]. To retain their structural and functional integrity, these components are necessary for organelles such as mitochondria and chloroplasts. Zn also aids in the reduction in oxidative stress caused by ROS species, resulting in an increase in the activity of antioxidants such as POD, SOD, APX, and CAT [39]. Ti is a mineral that is advantageous to plant development. When plants are deficient in Fe, Ti aids in the induction of genes involved in Fe acquisition, boosting Fe absorption and utilization and, as a result, aiding plant development.

The membrane damage produced via oxidative stress caused by Cd toxicity is shown through a significant drop in RWC and photosynthetic pigments and an increase in EL and MDA levels in Cd-exposed maize. Lipid peroxidation of the membrane allows ethylene, a stress hormone, to enter and disrupt the operation of organelles, such as the photosynthetic machinery. As a result, the chlorophyll content is reduced. Moreover, chlorophyll is essential for the synthesis of various organic substances in plants.

In this study, antioxidants and proline levels were observed to be higher in Cd-stressed plants. Plant defense mechanisms were harmed, resulting in decreased growth and yield. SOD, POD, CAT, APX, and proline aid plants in stress by scavenging and osmoregulating reactive oxygen species (ROS) [32]. By creating more H_2_O_2_, SOD protects the plant from oxidative-stress-related death. A higher level of SOD protects the plant against oxidative damage [40]. The conversion of H_2_O_2_ to H_2_O occurs in CAT. Another enzyme known as POD aids in detoxifying HM [41,42]. In Cd-stressed maize, all treatments, especially the combined treatment with PGPR and NPs, increased the activities of CAT, APX, POD, and proline.

In our in silico analysis, ZnO positively regulated peptidase M14 (BPUM_0711) and uncharacterized protein YqgT (yqgT), which are involved in metallocarboxypeptidase activity. ZnO activates trehalose-6-phosphate hydrolase (treA), which participates in the catabolic processes of alpha-phosphotrehalase. TiO_2_ positively regulates glutaminase (glsA), Cctalase (katX1), and catalase (katX2), which contribute to glutamine metabolic processes and oxidoreductase reactions. TiO_2_ negatively regulates glutamine synthetase (glnA), which induces the glutamate-ammonia ligase process and the cell response towards nitrogen homeostasis. ZnO positively regulates the Zn-dependent exopeptidase superfamily protein (GRMZM2G006069_P01) involved in metallocarboxypeptidase activity. TiO_2_ positively regulates catalase isozyme 1 (CAT1), catalase isozyme 2 (CAT2), and catalase isozyme 3 (CAT3), which are core regulators, which protect cellular machinery from the toxic effects of hydrogen peroxide. TiO_2_ positively regulates nucleic acid binding protein (GRMZM2G456132_P01) and DNA-damage-repair/toleration protein (GRMZM2G121311_P01), which are active participants in the oxidative demethylation process and the repair of damaged sites. TiO_2_ positively regulates the hydroxyproline-rich glycoprotein family protein (pco147635a), which is involved in demethylation and mRNA metabolism. 

The ZnO-TiO_2_ complex importantly triggered the biosynthesis of carbohydrates, speeding up the generation of the energy requirements in *B. pumilus*. On the other hand, the ZnO-TiO_2_ complex produced an additive effect in terms of stress management, affecting the machinery of cell defense proteins in maize.

## 5. Conclusions

In this study, we concluded that *B. pumilus* and ZnO and TiO_2_ NPs can reduce the symptoms caused by Cd stress in maize. Under conditions of Cd stress, *B. pumilus* and ZnO and TiO_2_ NPs successfully enhanced maize’s biomass, antioxidants, proline, nutritional content, and phytohormones. In maize, combining *B. pumilus* with NPs resulted in the maximum growth and improved defensive mechanisms against Cd stress. Furthermore, PGPR and metallic NPs showed synergistic effects, with strengthened performance as plant growth promoters. We concluded that using *B. pumilus* and ZnO and TiO_2_ NPs to reduce Cd stress in maize is a viable, environmentally friendly solution. Our in silico pipeline presented a novel picture of ZnO–TiO_2_ protein interaction and participation in both *B. pumilus* and maize. In this scenario, the ZnO–TiO_2_ combination is an ideal therapeutic strategy to encounter heavy metals, specifically Cd, in order to combat their effects on plant growth and physiology. We also suggest that ZnO–TiO_2_ protein interactors could be excellent biomarkers of Cd toxicity.

## Figures and Tables

**Table 1:** Germination and biomass studies of *Zea mays* L. seedlings treated with PGPR.

T	SL (cm)	RL (cm)	LA (cm)^2^	FW (g)	DW (g)	GP	GI	PI	SVI
**C**	5.2 ^AB^ ± 0.02	2.2 ^A^ ± 0.02	2.1 ^AB^ ± 0.04	0.3 ^B^ ± 0.02	0.02 ^BC^ + 0.02	40 ^AB^ ± 0.03	1.2 ^AB^ ± 0.04	2.4 ^AB^ ± 0.02	1.4 ^BC^ ± 0.05
**JW**	6.9 ^A^ ± 0.02	6.5 ^A^ ± 0.03	5.3 ^A^ ± 0.03	1.2 ^A^ ± 0.01	0.14 ^A^ + 0.03	85.3 ^A^ ± 0.05	2.48 ^A^ ± 0.05	5.2 ^A^ ± 0.03	6.6 ^A^ ± 0.04
**13RKI**	3.1 ^AB^ ± 0.03	3.5 ^A^ ± 0.02	1.6 ^AB^ ± 0.04	0.49 ^B^ ± 0.07	0.02 ^AB^ + 0.04	53.3 ^BC^ ± 0.04	1.2 ^BC^ ± 0.07	3.4 ^BC^ ± 0.04	2.1 ^B^ ± 0.03
**GW3**	1.1 ^B^ ± 0.02	3.2 ^AB^ ± 0.02	1.13 ^AB^ ± 0.06	0.42 ^B^ ± 0.04	0.03 ^BC^ + 0.03	36.0 ^C^ ± 0.03	1.2 ^C^ ± 0.08	2.5 ^BC^ ± 0.01	1.1 ^BC^ ± 0.02
**BG**	3.4 ^AB^ ± 0.06	3.2 ^AB^ ± 0.02	1.9 ^AB^ ± 0.07	0.47 ^B^ ± 0.03	0.06 ^BC^ + 0.04	58.0 ^BC^ ± 0.01	1.1 ^C^ ± 0.08	3.4 ^BC^ ± 0.06	1.2 ^BC^ ± 0.03
**CM5**	1.4 ^B^ ± 0.02	3.1 ^AB^ ± 0.07	1.3 ^AB^ ± 0.04	0.49 ^B^ ± 0.01	0.06 ^BC^ + 0.03	52.2 ^C^ ± 0.02	1.2 ^C^ ± 0.05	3.1 ^C^ ± 0.07	2.1 ^BC^ ± 0.07
**RHG**	1.7 ^B^ ± 0.02	1.9 ^B^ ± 0.04	0.6 ^B^ ± 0.04	0.36 ^B^ ± 0.02	0.03 ^C^ + 0.05	61.3 ^BC^ ± 0.02	1.3 ^BC^ ± 0.07	3.7 ^BC^ ± 0.02	1.28 ^C^ ± 0.06
**MRH1**	3.2 ^AB^ ± 0.09	3.3 ^AB^ ± 0.02	1.2 ^AB^ ± 0.09	0.36 ^B^ ± 0.03	0.03 ^BC^ + 0.05	62 ^BC^ ± 0.04	1.2 ^C^ ± 0.04	3.2 ^BC^ ± 0.04	0.5 ^C^ ± 0.09
**RH10**	2.0 ^B^ ± 0.10	3.1 ^AB^ ± 0.10	1.2 ^AB^ ± 0.10	0.22 ^B^ ± 0.03	0.03 ^BC^ + 0.06	46.6 ^C^ ± 0.03	1.1 ^C^ ± 0.02	3.2 ^C^ ± 0.12	1.1 ^BC^ ± 0.10
**9K**	1.2 ^AB^ ± 0.02	3.6 ^A^ ± 0.10	1.9 ^AB^ ± 0.08	0.34 ^B^ ± 0.10	0.04 ^BC^ + 0.11	40.1 ^C^ ± 0.2	0.8 ^C^ ± 0.12	2.7 ^C^ ± 0.22	1.2 ^BC^ ± 0.05
**8RK1**	1.3 ^AB^ ± 0.12	1.7 ^A^ ± 0.12	0.5 ^B^ ± 0.11	0.22 ^AB^ ± 0.05	0.05 ^BC^ + 0.11	53.3 ^BC^ ± 0.44	0.5 ^C^ ± 0.11	2.7 ^BC^ 0.11	1.1 ^BC^ ± 0.06

Data represent mean values ± standard error (SE) of three replicates. Each mean value followed by a specific letter represents a significant difference at *p* < 0.05, generated through Statistix 8.1.

**Table 2:** Germination and biomass studies of *Zea mays* L. treated with different concentrations of TiO_2_ NPs (10, 20, and 30 ppm) in a Petri plate experiment.

T	SL	RL	LA	FW	DW	GP	GI	PI	SVI
**C**	2.4 ^B^ ± 0.06	1.5 ^B^ ± 0.07	0.89 ^C^ + 0.07	0.27 ^B^ + 0.06	0.05 ^AB^ + 0.04	63 ^B^ + 0.02	1.5 ^B^ + 0.07	2.6 ^B^ + 0.07	2.4 ^C^ + 0.07
**10 (PPM)**	4.6 ^AB^ ± 0.03	3.8 ^AB^ ± 0.02	1.8 ^B^ + 0.03	0.73 ^AB^ + 0.02	0.03 ^AB^ + 0.02	73 ^AB^ + 0.08	1.8 ^AB^ + 0.02	3.01 ^AB^ + 0.02	4.6 ^AB^ + 0.02
**20 (PPM)**	5.1 ^A^ ± 0.04	4.4 ^A^ ± 0.08	2.5 ^A^ + 0.04	0.14 ^A^ + 0.08	0.09 ^A^ + 0.08	83 ^A^ + 0.04	2 ^A^ + 0.08	3.9 ^A^ + 0.08	5.1 ^A^ + 0.08
**30 (PPM)**	1.2 ^BC^ ± 0.07	1.1 ^B^ ± 0.02	0.4 ^C^ + 0.02	0.4 ^B^ + 0.02	0.04 ^AB^ + 0.02	76 ^AB^ + 0.08	1.9 ^AB^ + 0.08	2.03 ^BC^ + 0.02	2.2 ^C^ + 0.02

Data represent mean values ± standard error (SE) of three replicates. Each mean value followed by a specific letter represents a significant difference at *p* < 0.05, generated through Statistix 8.1.

**Table 3:** Germination and biomass studies of *Zea mays* L. treated with different concentrations of ZnO NPs (10, 20, and 30 ppm) in a Petri plate experiment.

T	SL	RL	LA	FW	DW	GP	GI	PI	SVI
**C**	1.9 ^B^ ± 0.08	1.1 ^B^ ± 0.02	0.44 ^C^ + 0.02	0.18 ^BC^ + 0.03	0.04 ^B^ + 0.05	60.2 ^BC^ + 0.04	1.18 ^B^ + 0.09	2.18 ^AB^ + 0.06	2.1 ^B^ + 0.08
**10 (PPM)**	4.1 ^AB^ ± 0.04	3.2 ^AB^ ± 0.03	1.44 ^B^ + 0.05	0.11 ^BC^ + 0.05	0.02 ^C^ + 0.07	71.1 ^B^ + 0.09	1.13 ^B^ + 0.10	2.11 ^AB^ + 0.07	4.1 ^AB^ + 0.02
**20 (PPM)**	4.9 ^A^ ± 0.03	4.1 ^A^ ± 0.04	2.1 ^A^ + 0.05	0.82 ^A^ + 0.04	0.08 ^A^ + 0.09	81.2 ^A^ + 0.02	2.22 ^A^ + 0.08	3.7 ^A^ + 0.04	4.8 ^A^ + 0.08
**30 (PPM)**	1.1 ^C^ ± 0.03	0.9 ^BC^ ± 0.03	0.3 ^C^ + 0.05	0.09 ^C^ + 0.05	0.03 ^BC^ + 0.10	74.3 ^AB^ + 0.07	1.1 ^B^ + 0.09	1.2 ^B^ + 0.05	1.1 ^C^ + 0.02

Data represent mean values ± standard error (SE) of three replicates. Each mean value followed by a specific letter represents a significant difference at *p* < 0.05, generated through Statistix 8.1.

**Table 4:** Biomass estimations of *Zea Mays* L. plants treated with PGPR and TiO_2_ and ZnO NPs under Cd stress (120 ppm).

T	SL (cm)	RL (cm)	LA (cm)^2^	FW (g)	RWC (g)	DW (g)	GP	GI	PI	SVI
**C**	14.2 ^AB^ ± 0.02	5.8 ^B^ ± 0.01	13.2 ^B^ ± 0.01	5.1 ^B^ ± 0.02	13.2 ^AB^ ± 0.03	1.85 ^AB^ ± 0.03	68.2 ^AB^ ± 0.03	2.1 ^AB^ ± 0.04	2.3 ^AB^ ± 0.02	16.2 ^AB^ ± 0.03
**Cd Stress**	8.9 ^B^ ± 0.02	3.1 ^B^ ± 0.02	5.7 ^C^ ± 0.02	2.4 ^BC^ ± 0.01	7.1 ^B^ ± 0.05	0.66 ^B^ ± 0.05	51.2 ^B^ ± 0.05	0.6 ^B^ ± 0.05	0.5 ^B^ ± 0.03	4.3 ^B^ ± 0.05
**TiO_2_ NPs**	30.1 ^AB^ ± 0.03	11.8 ^AB^ ± 0.05	14.9 ^AB^ ± 0.04	7.7 ^AB^ ± 0.07	16.3 ^AB^ ± 0.04	2.1 ^AB^ ± 0.04	61.3 ^AB^ ± 0.04	2.2 ^A^ ± 0.07	3.1 ^AB^ ± 0.04	18.3 ^AB^ ± 0.04
**TiO_2_ NPs + Cd**	22.5 ^AB^ ± 0.02	9.1 ^AB^ ± 0.06	14.6 ^AB^ ± 0.06	7.1 ^AB^ ± 0.04	15.0 ^AB^ ± 0.03	1.2 ^AB^ ± 0.03	75.0 ^AB^ ± 0.03	1.5 ^AB^ ± 0.08	2.1 ^AB^ ± 0.01	17 ^AB^ ± 0.03
**PG**	31 ^A^ ± 0.06	13 ^AB^ ± 0.04	14.8 ^AB^ ± 0.07	7.47 ^AB^ ± 0.03	22.2 ^AB^ ± 0.01	2.3 ^AB^ ± 0.01	73.0 ^AB^ ± 0.01	2.6 ^A^ ± 0.08	3.1 ^AB^ ± 0.06	24.2 ^AB^ ± 0.01
**PG + Cd**	23.2 ^AB^ ± 0.02	11.2 ^AB^ ± 0.09	13.2 ^AB^ ± 0.04	6.49 ^AB^ ± 0.01	15 ^AB^ ± 0.02	2.2 ^AB^ ± 0.02	72 ^AB^ ± 0.02	1.6 ^AB^ ± 0.05	3.2 ^AB^ ± 0.07	15 ^AB^ ± 0.02
**TiO_2_ NPs + PG**	42.2 ^A^ ± 0.02	21 ^A^ ± 0.02	33 ^A^ ± 0.04	8.36 ^A^ ± 0.02	24 ^A^ ± 0.01	4.2 ^A^ ± 0.02	82 ^A^ ± 0.02	3.1 ^A^ ± 0.07	4.1 ^A^ ± 0.02	27 ^A^ ± 0.02
**TiO_2_ NPs + PG + Cd**	31.4 ^AB^ ± 0.09	18.2 ^AB^ ± 0.05	29 ^AB^ ± 0.05	7.36 ^AB^ ± 0.03	14 ^AB^ ± 0.02	3.2 ^AB^ ± 0.02	71 ^AB^ ± 0.04	2.5 ^AB^ ± 0.04	3.1 ^AB^ ± 0.04	17 ^AB^ ± 0.04
**ZnO NPs**	28.2 ^AB^ ± 0.10	11 ^AB^ ± 0.10	14.7 ^AB^ ± 0.10	7.22 ^AB^ ± 0.03	15 ^AB^ ± 0.02	2.2 ^AB^ ± 0.02	63 ^AB^ ± 0.03	2.2 ^AB^ ± 0.02	3.6 ^AB^ ± 0.12	18 ^AB^ ± 0.03

Data represent mean values ± standard error (SE) of three replicates. Each mean value followed by a specific letter represents a significant difference at *p* < 0.05, generated through Statistix 8.1.

**Table 5:** Results of nutrient analysis of Cd stress, showing a decrease in the values of Fe, Mg, and Cu, as compared to control and treated plants. Values of Ca, Z, k, Cd, and Na were increased compared to controls but decreased in comparison to the treated plants.

T	Cu	Ca	Zn	K	Mg	Cd	Fe	Na
**C**	11.310 ^A^ ± 0.02	1.53 ^A^ ± 0.02	0.310 ^A^ ± 0.02	1 ^AB^ ± 0.02	5.350 ^A^ ± 0.02	1 ^A^ ± 0.02	4.621 ^A^ ± 0.02	1 ^A^ ± 0.02
**Cd Stress**	8.9 ^B^ ± 0.02	3.1 ^B^ ± 0.02	5.7 ^C^ ± 0.02	2.4 ^BC^ ± 0.01	2.1 ^B^ ± 0.05	5.66 ^B^ ± 0.05	1.2 ^B^ ± 0.05	2.6 ^B^ ± 0.05
**TiO_2_ NPs + Cd**	22.5 ^AB^ ± 0.02	9.1 ^AB^ ± 0.06	14.6 ^AB^ ± 0.06	7.1 ^AB^ ± 0.04	15.0 ^AB^ ± 0.03	5.2 ^AB^ ± 0.03	75.0 ^AB^ ± 0.03	1.5 ^AB^ ± 0.08
**PG + Cd**	23.2 ^AB^ ± 0.02	11.2 ^AB^ ± 0.09	13.2 ^AB^ ± 0.04	6.49 ^AB^ ± 0.01	15 ^AB^ ± 0.02	5.4 ^AB^ ± 0.02	72 ^AB^ ± 0.02	1.6 ^AB^ ± 0.05
**TiO_2_ NPs + PG + Cd**	31.4 ^AB^ ± 0.09	18.2 ^AB^ ± 0.05	29 ^AB^ ± 0.05	7.36 ^AB^ ± 0.03	14 ^AB^ ± 0.02	3.2 ^AB^ ± 0.02	71 ^AB^ ± 0.04	2.5 ^AB^ ± 0.04
**ZnO NPs + PG + Cd**	32 ^AB^ ± 0.12	12 ^AB^ ± 0.11	30 ^AB^ ± 0.14	6.22 ^AB^ ± 0.05	24.3 ^AB^ ± 0.11	3.9 ^AB^ ± 0.08	62.3 ^AB^ ± 0.44	2.2 ^AB^ ± 0.11

Data represent mean values ± standard error (SE) of three replicates. Each mean value followed by a specific letter represents a significant difference at *p* < 0.05, generated through Statistix 8.1.

## Data Availability

Data will be available on request to the corresponding or first author. The data are not publicly available due to this is a part of the Ph.D thesis and not completed yet.
