# Correction: Shafiq et al. Titanium Oxide and Zinc Oxide Nanoparticles in Combination with Cadmium Tolerant *Bacillus pumilus* Ameliorates the Cadmium Toxicity in Maize. *Antioxidants* 2022, *11*, 2156

**DOI:** 10.3390/antiox14030316

**Published:** 2025-03-06

**Authors:** Tayyab Shafiq, Humaira Yasmin, Zafar Abbas Shah, Asia Nosheen, Parvaiz Ahmad, Prashant Kaushik, Ajaz Ahmad

**Affiliations:** 1 Department of Biosciences, COMSATS University Islamabad (CUI), Islamabad 45550, Pakistan; tayyabshafiqe@gmail.com (T.S.); asianosheen@yahoo.com (A.N.); 2 Department of Bioinformatics, Hazara University, Mansehra 21120, Pakistan; zafarabbasshah92@gmail.com; 3 Department of Botany, GDC Pulwama, Pulwama 192301, Jammu and Kashmir, India; 4 Independent Researcher, 46022 Valencia, Spain; prashantumri@gmail.com; 5 Department of Clinical Pharmacy, College of Pharmacy, King Saud University, Riyadh 11451, Saudi Arabia; ajukash@gmail.com

In the original publication [1], there was an error regarding the affiliation for author Prashant Kaushik. Specifically, the fourth affiliation (Instituto de Conservación y Mejora de la Agrodiversidad Valenciana, Universitat Politècnica de València, 46022 Valencia, Spain) has been removed from the affiliation list, as per the institution’s request. Dr. Prashant Kaushik should have been listed as an Independent Researcher. The fourth affiliation of Prashant Kaushik should have been be as follows:

Independent Researcher, 46022 Valencia, Spain; prashantumri@gmail.com

The institutional email for the author associated with the institution has also been changed with another email.

The authors state that the scientific conclusions are unaffected. This correction was approved by the Academic Editor. The original publication has also been updated.
